# Phylogenetic Exploration of Nosocomial Transmission Chains of 2009 Influenza A/H1N1 among Children Admitted at Red Cross War Memorial Children’s Hospital, Cape Town, South Africa in 2011

**DOI:** 10.1371/journal.pone.0141744

**Published:** 2015-11-13

**Authors:** Ziyaad Valley-Omar, Fredrick Nindo, Maanda Mudau, Marvin Hsiao, Darren Patrick Martin

**Affiliations:** 1 Centre for Respiratory Diseases and Meningitis, Virology, National Institute for Communicable Diseases, Sandringham, Johannesburg, South Africa; 2 University of Cape Town, Faculty of Health Sciences, Department of Clinical Laboratory Sciences Medical Virology, Observatory, Cape Town, South Africa; 3 University of Cape Town, Faculty of Health Sciences, Institute of Infectious Disease and Molecular Medicine, Computational Biology Group, Observatory, Cape Town, South Africa; 4 Centre for Tuberculosis, National Institute for Communicable Diseases, Sandringham, Johannesburg, South Africa; 5 National Health Laboratory Service, Groote Schuur Complex, Department of Clinical Virology, Observatory, Cape Town, South Africa; Centers for Disease Control and Prevention, UNITED STATES

## Abstract

Traditional modes of investigating influenza nosocomial transmission have entailed a combination of confirmatory molecular diagnostic testing and epidemiological investigation. Common hospital-acquired infections like influenza require a discerning ability to distinguish between viral isolates to accurately identify patient transmission chains. We assessed whether influenza hemagglutinin sequence phylogenies can be used to enrich epidemiological data when investigating the extent of nosocomial transmission over a four-month period within a paediatric Hospital in Cape Town South Africa. Possible transmission chains/channels were initially determined through basic patient admission data combined with Maximum likelihood and time-scaled Bayesian phylogenetic analyses. These analyses suggested that most instances of potential hospital-acquired infections resulted from multiple introductions of Influenza A into the hospital, which included instances where virus hemagglutinin sequences were identical between different patients. Furthermore, a general inability to establish epidemiological transmission linkage of patients/viral isolates implied that identified isolates could have originated from asymptomatic hospital patients, visitors or hospital staff. In contrast, a traditional epidemiological investigation that used no viral phylogenetic analyses, based on patient co-admission into specific wards during a particular time-frame, suggested that multiple hospital acquired infection instances may have stemmed from a limited number of identifiable index viral isolates/patients. This traditional epidemiological analysis by itself could incorrectly suggest linkage between unrelated cases, underestimate the number of unique infections and may overlook the possible diffuse nature of hospital transmission, which was suggested by sequencing data to be caused by multiple unique introductions of influenza A isolates into individual hospital wards. We have demonstrated a functional role for viral sequence data in nosocomial transmission investigation through its ability to enrich traditional, non-molecular observational epidemiological investigation by teasing out possible transmission pathways and working toward more accurately enumerating the number of possible transmission events.

## Introduction

Despite the existence of infection control policies and protocols, nosocomial transmission of respiratory viruses is a common problem that can occur in virtually any health-care setting [1–7]. The close proximity of patients, visitors and healthcare workers (HCWs) to one another, virus shedding during asymptomatic periods, low vaccination compliance (by both HCWs and the general public) as well as virus persistence in respiratory secretions and fomites can all contribute to the spread of the virus and lead to nosocomial outbreaks [2, 8–12]. Limiting nosocomial transmissions in hospitals is particularly important as besides increasing the duration and costs of hospitalization, they can increase morbidity and mortality, particularly in high-risk elderly, infant, seriously ill, and immunocompromised patients [2, 3, 13].

Traditionally, investigation of influenza nosocomial transmission has required a combination of confirmatory influenza diagnostic testing (usually PCR) and epidemiological investigation. PCR-based molecular diagnostic assays are generally limited in their capacity to classify etiologic agents beyond the type/subtype level. While this is acceptable for the diagnosis of uncommon hospital acquired infections (HAIs), for more common causes of HAIs such as influenza it is desirable to apply assays that yield enough data to more accurately distinguish unique patient transmission chains. In conjunction with epidemiological data such assays could be effectively used to test whether patient infections have a nosocomial origin [6, 14–17].

Continued innovation in pathogen sequencing techniques have enabled the rapid and cost-effective generation of greater volumes of molecular genetic data from routine diagnostic assays [18]. For rapidly evolving pathogens such as RNA viruses which accumulate high degrees of genetic diversity during the course of an epidemic, genetic data has been used to provide valuable epidemiological insights [19–24]. Specifically, the marriage of phylogenetics, population genetics and epidemiology within the fledgling field of phylodynamics enables the use of nucleotide sequence data and clinical history to study disease transmission dynamics. This form of molecular epidemiology has become a particularly useful adjunct to clinical histories when attempting to determine the nature and timing of infections [25–33].

Recently, influenza nosocomial transmission investigations have employed molecular techniques to corroborate comprehensive epidemiological data sets [4–7, 34]. These studies through the sequencing of the Influenza hemagglutinin (HA) /neuraminidase (NA) and/or PB2 genomic regions, enabled researchers to determine virus isolate phylogenies and the potential epidemiological linkages between cases and HCWs. This was accomplished by the reconstruction of viral phylogenies using Maximum Parsimony, Maximum Likelihood or Neighbour Joining methods. While these studies could effectively support epidemiological data by linking cases through “identical viruses” they were unable to gauge or ascertain epidemiological linkage when viruses have minor sequence variations.

The potential analytical power afforded by being able to use sequence data to accurately infer close epidemiological linkages has been highlighted in large-scale Influenza sequencing studies that identified sequence variations that arose within single individuals during infection and were then transmitted to multiple individuals residing in the same household/hospital ward [35–38]. The continuing development of increasingly sophisticated molecular evolutionary analysis tools [22, 39–41] is improving our ability to accurately determine, in almost real-time, key epidemiological variables such as the basic reproduction rate (R_0_) [21, 39, 42–47].

Among the most commonly applied of these new tools are those implemented in the computer program Bayesian Evolutionary Analysis Sampling Trees (BEAST) [48]. The Bayesian framework upon which BEAST has been built enables statistically rigorous testing of alternative evolutionary hypotheses relating to the emergence and spread of infectious diseases using a combination of sequence data and data related to the sequences such as either the locations and times when they were sampled, or the clinical features of the diseases that they were associated with [49, 50]. Besides enabling the construction of time-scaled phylogenies (as opposed to the more usual genetic-distance scaled variety), BEAST analyses can identify spatial and temporal linkages between viruses which, along with clinical case records, can be used to confidently identify transmission chains within broader transmission networks [39, 51–54].

Despite these notable analytical advances in recent years only a few studies have investigated influenza nosocomial transmission at the nucleotide level. Most nosocomial infection studies have simply focused on observing patient Influenza like illness (ILI) symptoms and/or conducted confirmatory influenza molecular tests. In South Africa there is no reliable information available on the frequency of nosocomial respiratory virus transmissions. There is similarly sparse information on the proportions of HCWs who are vaccinated against influenza and other respiratory viruses. It is, however, assumed that vaccine compliance is low amongst South African HCWs, as is the worldwide trend, which is concerning given that increased influenza vaccine coverage amongst HCW correlates with decreased incidence of ILI in hospitalised patients [55–57].

In order to implement a cost effective vaccination strategy and evaluate the effectiveness of infection control procedures in an environment where influenza nosocomial transmission is assumed to be common, the burden of nosocomial disease needs to be evaluated. Here we assess whether an influenza sequence phylogenetic analysis could, by enriching basic epidemiological data, assist with investigating transmission chains and the frequency of nosocomial transmission within Red Cross War Memorial Childrens Hospital (RXH) in Cape Town South Africa. Over a 4-month period, influenza A positive patient samples from various wards in the hospital were obtained and partial influenza hemagglutinin sequences were generated for each patient. Possible transmission chain/channels were determined through a combination of basic patient admission data and the analysis of Maximum likelihood and time-scaled Bayesian phylogenetic trees, to identify potentially epidemiologically linked transmission chains. The inclusion of a time-scaled phylogenetic analysis allowed us to estimate times to most recent common ancestors of epidemiologically and phylogenetically linked samples with statistical support to evaluate the likelihood of phylogenetically related infections sharing a common transmission source.

## Materials and Methods

### Study samples

**Fig 1:**
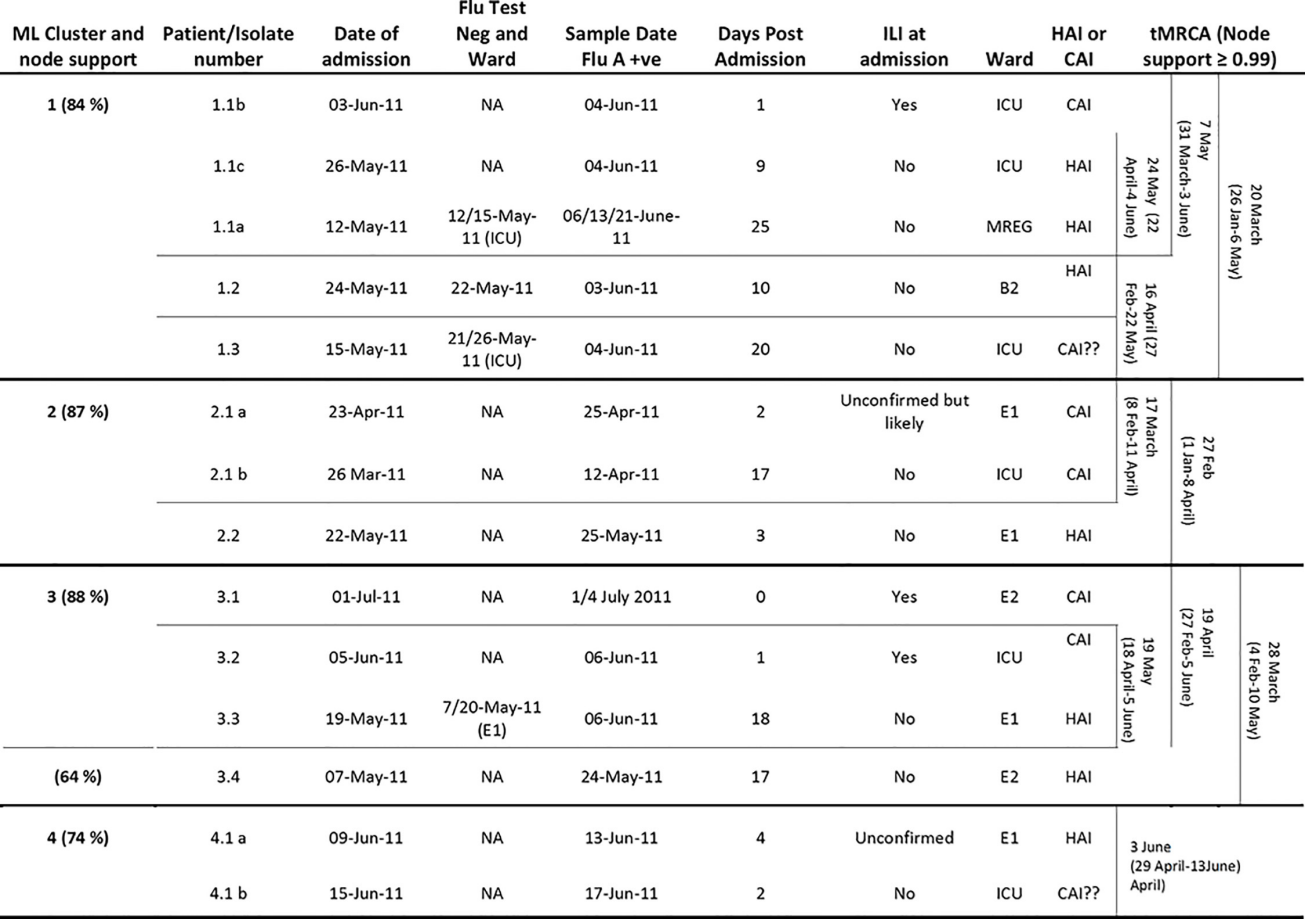
RXH patient admission, Flu positivity and assumed source of viral acquisition (HAI/CAI) cases/viral isolate data grouped according to viral sequence phylogeny.

Archived influenza positive patient RNA samples were obtained from the National Health Laboratory Service (NHLS) within Groote Schuur Hospital in Cape Town, South Africa, which routinely conducts respiratory virus diagnostic PCR testing for surrounding public hospitals including RXH using the Seeplex ® RV7 respiratory virus detection system (Seegene). RXH is the largest tertiary paediatric hospital in South Africa and manages approximately 260 000 patient visits per year. Most of its patients are from poor communities, a third of which are less than one year old. Although > 98% of patients are referred to the hospital from the Western Cape region of South Africa, referrals from the rest of South Africa are also common as a result of the unique and high level of paediatric care available at the hospital. For the period spanning 1 April to 31 July 2011, a total of eighteen samples were obtained. Fourteen were confirmed Influenza A, pandemic H1N1 (H1N1pdm) positive RNA samples from pediatric patients admitted to RXH, which formed the focus of our study. A further four samples, served as geographic controls and were obtained from three other hospitals within the same region during the same time period; 1 from Groote Schuur Hospital (GRS), 1 from 2-Military Hospital (2MIL) and 2 from Somerset Hospital (SOM). Clinical history and hospital admission dates were abstracted from hospital records of the RXH patients from whom virus samples were obtained. This study was approved by the University of Cape Town Human Research Ethics Committee (UCT research ethics number: 305/2012). To ensure patient anonymity, study samples were unlinked by being issued with a study identification number. For the purpose of this investigation the transmission case definitions were as follows: (1) a suspected hospital-acquired influenza infection (HAI) case was defined as an influenza PCR positive individual who did not present to the hospital with respiratory symptoms or was confirmed to be influenza negative by laboratory testing within three days of admission (Fig 1); and (2) a suspected community-acquired infection (CAI) case was defined as a patient with laboratory-confirmed Influenza A (H1N1pdm) at or within three days of admission.

### PCR amplification and sequencing

cDNA was synthesized using a random hexamer primer set with the RevertAid First Strand cDNA Synthesis Kit (Thermo Scientific) according to manufacturer’s instructions. All influenza A genomic segments were amplified using a multisegment PCR with the universal primer set, MBTuni-12 (5’-ACGCGTGATCAGCAAAAGCAGG-3’) and MBTuni-13 (5’-ACGCGTGA TCAGTAGAAACAAGG-3’) and PCR conditions adapted from the RT-PCR method previously described [58]. Influenza A, H1N1pdm09 Gene-specific primers HA(H1) (5’-ACRTGTTACCCAGGRGATTTC-3’) and HA(H1) (5’-TCTTTACCYACTRCTGTGAA-3’) were used to amplify a partial segment of the H1N1pdm hemagglutinin gene (379–1204) as in the World Health Organization Influenza Sequencing Primers and Protocol (2009). PCR products were verified on a 1% agarose gel and directly sequenced using the ABI PRISM dye terminator cycle-sequencing kit V3.1 (Applied Biosystems) with the HA(H1) F and R primer set.

### Phylogenetic analysis

Potential nosocomial transmission clusters were identified by examining the phylogenetic relationships among the sequences in our dataset. Fourteen HA sequences from RXH and four geographic controls from surrounding hospitals were analysed together with homologous H1N1pdm HA sequences sampled from elsewhere in South Africa (n = 53) (GenBank accession numbers JF745847 to KT036481) during the same flu season and a representative set of sequences from other continents (n = 34), all available in GenBank (S1 Table, Accession numbers) (accessed on July 26 2013). These 123 sequences were aligned using MUSCLE with default settings [59]. Phylogenies were determined using the maximum likelihood (ML) and Bayesian Markov chain Monte Carlo (MCMC) approaches. The ML phylogenetic tree was constructed using RaxML [60] with the GTR-GAMMA nucleotide substitution model with branch support assessed with 1000 bootstrap replicates.

Genetic distances of aligned RXH HA amino sequences when compared to the 2011 H1N1 vaccine strain (A/California/7/2009 (H1N1)-like virus, accession number: KF527477) were determined using BioEdit Version 7.2.5.

Time-scaled Bayesian MCMC trees were constructed using BEAST available at (http://beast.bio.ed.ac.uk/) [41] and were used to approximate the dates of potential virus introduction events into RXH. We employed a GTR + G nucleotide substitution model and a coalescent constant population size demographic model with an initial H1N1pdm nucleotide substitution rate of 0.003 [21] assuming gamma distributed rates of nucleotide substitution. We ran two independent chains under relaxed clock models for 100 million generations sampling every 10000 generations. The runs were assessed for convergence and adequate mixing of model parameter estimates using Tracer (http://beast.bio.ed.ac.uk/Tracer). The resultant trees were summarised using Tree annotator (http://beast.bio.ed.ac.uk/TreeAnnotator) after discarding the first 10% of trees in the Marcov chain as burn-in. This summarisation process generated a maximum clade credibility (MCC) tree with branches supported by an associated posterior probability of > 0.95 considered as having significant statistical support. The ML and MCC trees were visualized using FigTree (http://beast.bio.ed.ac.uk/FigTree).

## Results

### Description of Cases and Hospital Layout

The influenza cases had been admitted to five different wards (B1; B2; E1; E2; ICU and MREG) at RXH. The study sample size was limited by the low number of influenza positive samples acquired from RXH during the study period (12 April- 1 July 2011). It is noteworthy that influenza detection between these dates fell within the beginning and peak of the influenza detection season for 2011 (epidemiologic weeks 15 to 26) in the Western Cape, as determined by the South African Viral Watch, Influenza Surveillance program (Bar Graph in S1 Fig, Viral Watch 2011 Western Cape Influenza detection)[61]. The RXH building complex consists of several buildings, most with multiple levels, each physically separated to limit nosocomial transmission between wards. Five of the six wards where cases were identified (B1, B2, E1, E2 and ICU) are all housed in the same building, where B1/B2 (different wings), E1/E2 (different wings) and ICU are located on different floors. The MREG, an admission ward, is located in a separate building.

Transmission case definitions identified nine suspected HAI cases and five CAI laboratory-confirmed H1N1pdm cases within the observed time period (April-July 2011). HAI cases were distributed across physically separated wards E1 (n = 3), E2 (n = 2), ICU (n = 3) and B2 (n = 1) while CAI cases were distributed across E1 (n = 1), ICU (n = 3) and MREG (n = 1).

The time-point post-admission at which patients were found to be H1N1pdm positive was assumed to be the earliest time-point when ILI symptoms were noticed by attending clinicians (Fig 1). The length of cut-off time/period could have left the investigation prone to lead-time bias, where some individuals who were in the early stages of community acquired infection, asymptomatic at admission only exhibited symptoms later following admission, could be systematically falsely classified as HAI cases.

### Phylogenetic Identification of Potential Transmission Clusters

**Fig 2:**
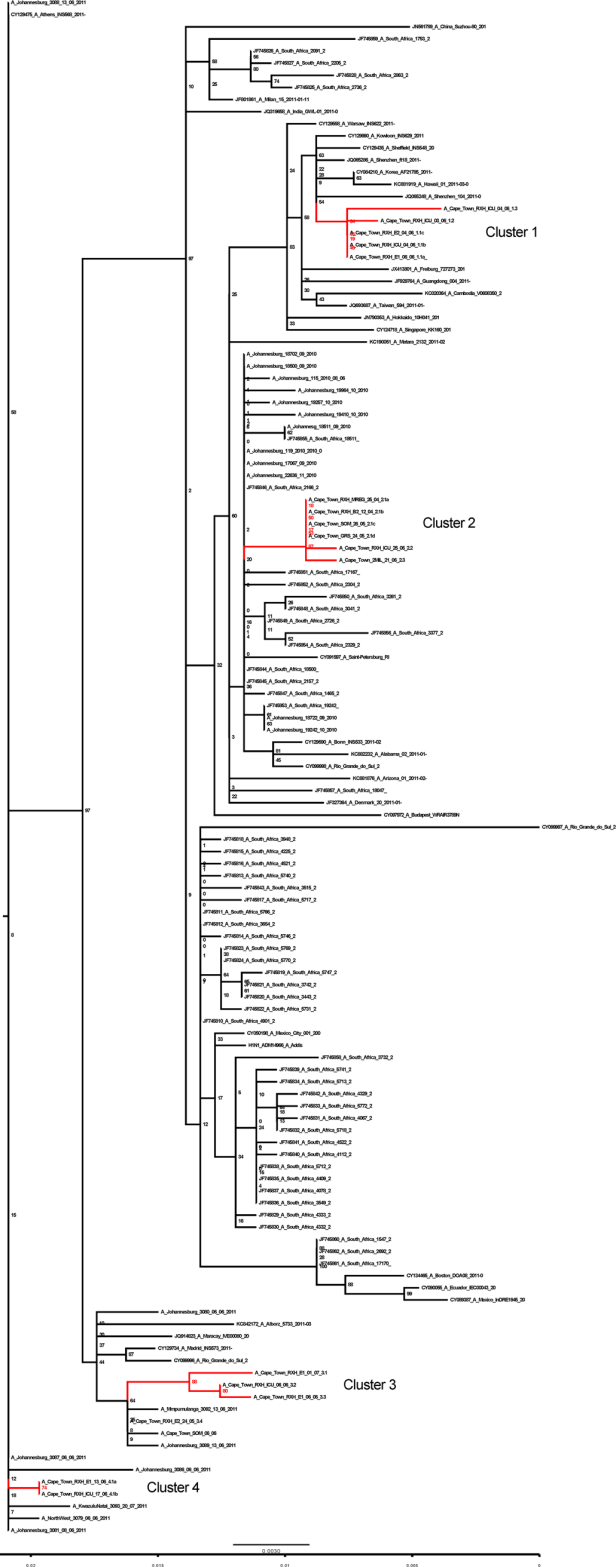
Maximum Likelihood tree constructed using RAxMLv8.0 under GTR GAMMA model of nucleotide evolution. Four highly supported (by bootstrap score) probable transmission clusters involving sequences isolated from the study site (RXH and surrounding facilities) are highlighted in red.

Nested PCR was used to generate partial Hemagglutinin (HA) amplicons spanning HA nucleotide positions 379 to 1204 (HA-1 sequence domain). Eighteen ~800 base pair amplicons were sequenced, aligned with 105 publically available H1N1pdm sequences (including all available South African sequences and sequences representative of the global H1N1 population in 2011) and used to construct a maximum likelihood tree. (Fig 2). In this tree, potential transmission clusters were defined as monophyletic clusters of two or more RXH sequences with more than 69% bootstrap support.

This analysis revealed four strongly supported (74–87% bootstrap support) potential RXH transmission clusters (clusters 1–4) containing 16 of the 18 Cape Town sequences (with 2 to 6 sequences in each cluster; Fig 2). These clusters appeared to have a temporal association rather than a geographical association. This is because the Cape Town RXH sequence clusters were interspersed with sequences from elsewhere in South Africa and the rest of the world. The RXH sequence clusters seem to be grouped according to the time-points at which the infections were identified. Furthermore, the degree of sequence diversity observed amongst the RXH isolates is amenable to differentiating instances of H1N1pdm HAI from instances of CAI.

### Transmission clusters using time-scaled phylogenies

**Fig 3:**
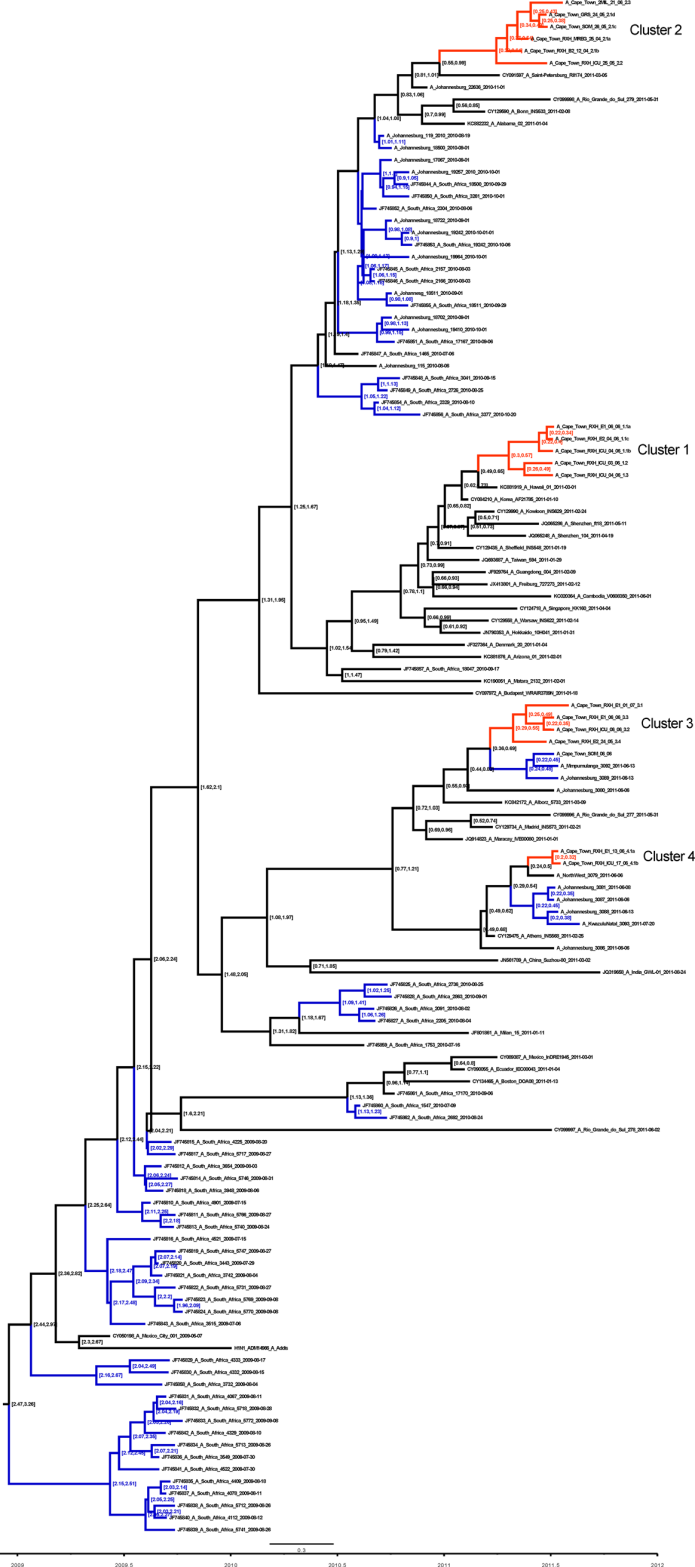
Time scaled maximum clade credibility (MCC) tree generated under GTR G + I and relaxed molecular clock in BEASTv1.7.5. The branch supports that are indicated are posterior probabilities (pp). Four significant putative transmission clusters containing sequences isolated from the study site (Red Cross Children’s Hospital and surrounding facilities) with pp support > 0.9 are highlighted in red.

To further explore the possibility that the four RXH clusters were the result of HAI, time-scaled Maximum Clade Credibility (MCC) phylogenies were constructed using BEAST. By the explicit inclusion of sampling times and estimated H1N1pdm evolutionary rates during the construction of the MCC tree, we were able to estimate the dates of the nodes in the tree. The dates of the root nodes of the four RXH clusters (and their 95% credibility intervals) were used to indicate whether they could plausibly have been the consequence of HAIs. Similar to the ML phylogenetic analysis, putative HAI transmission clusters were inferred if two or more sequences clustered with 0.95 or higher posterior probability support. Based on this criterion, exactly the same four RXH potential HAI transmission clusters were identified in the MCC tree (Fig 3) as were identified in the ML tree. Below we individually assess each of these clusters.

#### Cluster 1

Cluster 1 consists of five isolates sampled from individuals that were admitted to RXH, during a 22 day period between 12 May and 3 June 2011. Within a period of three days all of these individuals were confirmed by laboratory testing to have H1N1pdm infections (Figs 1, 2 and 3). Of these cases, four met the case definition of a HAI. Patient 1.1 b was the first patient to be confirmed H1N1pdm positive and also displayed ILI symptoms at admission and was thus classified as a CAI. Sequences derived from patients 1.1 c and 1.1 a were identical to those sampled from 1.1b and were confirmed H1N1pdm positive within 1 day of patient 1.1 b’s admission to the hospital but, due to the timing of these infections, it is unlikely that 1.1 c and 1.1 a acquired their infection from 1.1 b (Fig 2).

The other sequences in cluster 1: 1.2 and 1.3, differed from 1.1 a-c by one and three mutations, respectively. To determine the plausibility that the most recent common ancestor (MRCA) of all five of these sequences was present in RXH we estimated the date of the root node of this cluster. The date of this node was estimated, with 95% confidence to have been between the 26 January and 6 May 2011 with 26 March being the most probable date (Figs 1 and 3). This suggests that it is unlikely that all of the viruses in this cluster were transmitted within RXH as the 95% credibility interval of the time to most recent common ancestor (tMRCA) of these viruses excludes the admission dates of the patients from which the viruses were sampled (Fig 1).

The three identical sequences in this cluster 1.1 a–c have a mean estimated tMRCA of 7 May (range: 31 March-3 June), the upper limit of which coincides with the admission of patient 1.1 b, the presumed CAI infection in this cluster. 1.1 a and 1.1 c (the two probable HAI samples) have a mean estimated tMRCA of 24 May (range: 22 April-4 June), which is well within the period of admission of these patients implying that it is plausible that these viruses have a common hospital-derived source. While it is also plausible that patients 1.1 a and 1.1 c acquired their infections either directly or indirectly from a hospital source, due to the brief time lapse between of their diagnoses/symptoms relative to when patient 1.1b was admitted it is questionable whether these patients acquired their viruses from patient 1.1 b.

#### Cluster 2

Cluster 2 consists of six related sequences, three of which were sampled from RXH. Individuals 2.1 a (with a suspected CAI) and 2.1 b (with a suspected HAI) were infected with viruses that had identical HA sequences (Fig 2) both demonstrated H1N1pdm positive tests/ILI symptoms over a protracted period (~2 weeks apart). This would suggest that it is unlikely that these patients acquired their infections from one another or a common hospital source (Fig 1). This observation is supported by the finding that the 95% credibility interval of the tMRCA of these viruses excludes the admission dates of the patients from which the viruses were sampled. Suggesting that the potential HAI instance in this cluster may not share a common hospital ancestor with the other patients in the cluster and most likely was derived from different source.

#### Cluster 3

Cluster 3 consists of 3 unique sequences, all derived from RXH (3.1–3.3) (Fig 2). Patient 3.4 (Figs 2 and 3), also a RXH patient, has a sequence closely related to the sequences in cluster 3 but because of insufficient bootstrap support (< 69%) it was not considered as part of cluster 3. All patients whose viral isolates feature in cluster 3 (bootstrap supported) were admitted to RXH within a 43 day span of one another (19 May -1 July) and were confirmed to be H1N1pdm positive by laboratory testing within 25 days of one another. Viral isolates 3.1 and 3.2 are most likely to represent locally circulating CAIs as they tested positive for influenza A within 2 days of admission. Isolate 3.3, is the only sample to conform to our chosen criteria for a HAI in this cluster, testing H1N1pdm positive at 18 days post admission. The possibility does exist that patient 3.3 acquired their infection indirectly from patient 3.2 (the two patients were not housed in the same ward). The time-scaled phylogenetic analysis (Figs 1 and 3) may support this notion as it estimated that the virus isolates in patients 3.2 and 3.3, despite varying in sequence, may have shared a hospital ancestor between 18 April and 5 June (mean = 19 May) (Figs 1 and 3), the upper limit of which coincides with the co-hospitalisation of patients 3.2 and 3.3. In spite of this, due to time constraints, it is still highly unlikely that viral transmission occurred between these patients as the time-frame is an insufficient incubation period for infection symptoms. As indicated, insufficient bootstrap ML support precluded the sequence derived from patient 3.4 from cluster 3. Time-scaled phylogenetic analysis further confirmed that it is highly unlikely that 3.3 and 3.4 shared a common hospital ancestor since the 95% credibility interval of the tMRCA excluded the admission dates of both patients. Suggesting 2 different, unidentified hospital sources for these HAI instances.

#### Cluster4

Cluster 4 had two identical RXH-derived isolates (4.1 a and 4.1 b) isolated from RXH patients that were admitted six days apart to two different wards (E1 and ICU) and were confirmed as H1N1pdm positive by laboratory-testing within 4 days of one another, neither was observed to have ILI symptoms at admission. Patient 4.1 b qualified as having a potential CAI testing H1N1pdm positive two days post admission (admitted after Patient 4.1 a). Patient 4.1 a on the other hand had a suspected HAI and tested H1N1pdm positive at four days post admission. The MCC tree supports the inference that these infection instances are unlikely to have a common hospital source as the mean estimated tMRCA of 3 June (range: 29 April-13 June), the upper limit of which coincides with the admission of patient 4.1 a, the patient in this cluster with a likely CAI.

### Comparison with Epidemiological information

To determine the value of the insights derived from the influenza sequence analyses, we compared our interpretations to those derived from an epidemiological study conducted on a subset of our samples. Aside from an Influenza positive PCR, the epidemiological study used no other molecular techniques. Epidemiological observations was available for nine RXH patients (1.1 a; 1.1 b; 1.2; 1.3; 2.2; 3.2, 3.3; 4.1 a, 4.1 b) in 3 of the 4 clusters of interest (Clusters 1; 3 and 4) and are summarised in Fig 3 to show patient influenza positivity and movement within the hospital.

**Fig 4:**
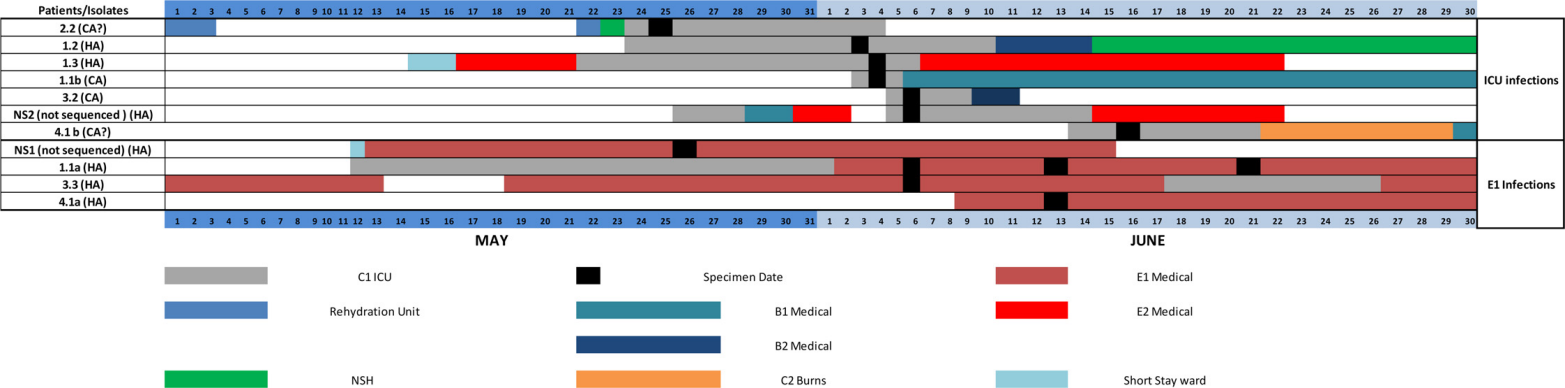
Timeline displaying patient admission, movement and Influenza A (H1N1pdm) positivity within hospital (patient/isolate identity according to ML Cluster identity).

Based on the molecularly indistinguishable Influenza instance data shown in Fig 4 (proximity and timing), which stratified infection instances according to ward, multiple HAI instances could have easily been assumed to have originated from a few identifiable index influenza A infected patients. This assumption of direct infection instance relatedness would be based on co-admission of patients to the same ward within a specific time-frame. This could incorrectly suggest transmission linkage between patients that were infected with viruses with MRCAs that excluded all possibility of a direct intra-hospital transmission chains. Incorrectly assuming transmission linkages between such patients, would lead to an underestimation of the number of times that influenza had been independently introduced into the hospital. While our sequence data suggests that there were at least four and potentially as many as ten independent H1N1pdm introductions into RXH between 26 March 2011 and 15 June 2011 due to no identifiable linkage between the HAI instances. The value of sequence data is thus highlighted by our ability to test the plausibility of assumed transmission pathways and attempt to more accurately enumerate the number of possible HAI and CAI transmission events.

## Discussion

This study demonstrated the value of including molecular sequence analysis as a diagnostic tool to identify nosocomial infections and trace the origins of viruses causing these infections. We have shown how such sequence data could enrich more traditional observational epidemiological data to reveal what may appear to be individual transmission chains but may in fact be multiple independent chains.

Unexpectedly, with the available data we were unable to conclusively identify patient sources of infection for any of the potential HAI instances identified (1.1 a; b; 2.1 b; 2.2; 3.3; 3.4 and 4.1 a). Furthermore, temporally-scaled phylogenetic analysis suggested that most instances of potential HAI in RXH—including instances where virus HA sequences were identical between different cases—likely resulted from independent introductions of Influenza A viruses into the hospital. Our inability to establish epidemiological transmission linkage of cases/viral isolates could be attributed to limited sampling in RXH during the study period which could imply that the viruses we detected originated in either asymptomatic hospital patients, visitors, or hospital staff. Transmission from asymptomatic patients is serious in the context of South Africa’s high HIV prevalence. While studies have demonstrated that HIV-infection status alone in adults does not increase risk of Influenza infection, evidence suggests that prolonged Influenza shedding periods (> 1 week) can be observed in patients exhibiting non-HIV related immunosuppression [62, 63]. Such prolonged influenza positivity was exhibited in patient 1.1 a, who remained influenza PCR positive for 15 days. In-spite of this, none of the RXH patients were HIV positive during the course of this study and thus could not account for prolonged ILI and possible influenza shedding in patient 1.1 a.

Furthermore, while we are also unable to confirm the vaccination status of patients, it is unlikely that they were vaccinated as the South African public sector is not provided with free access to flu vaccination. Flu vaccination via herd immunity may nevertheless have mitigated transmission from and between vaccinated adults. Approximately 75% of all influenza positive cases in South Africa during the 2011 influenza infection season were as a result of influenza A(H1N1)pdm09 virus infection [61]. The genetic distance between the 2011 Southern hemisphere H1N1 vaccine strain (A/California/7/2009 (H1N1)-like virus) and virus isolates derived from our patients (all of which were influenza A(H1N1) pdm09 positive) showed a mean similarity genetic distance of 0.975 (range 0.966–0.985) for the HA region analysed. It is also noted that the vast majority of influenza A(H1N1)pdm09 viruses characterized by the WHO Global Influenza Surveillance and Response System during the flu season in question were antigenically related to the above-mentioned influenza A(H1N1)pdm09-like virus included in the 2011 Southern hemisphere vaccine [61].

Nevertheless, the protective effect of herd immunity would be limited as influenza vaccination compliance in the general public and HCWs in South Africa is expected to be low. This occurs despite its potential to reduce morbidity in HCWs and limit transmission to patients, which might invaluably contribute to the maintenance of the workforce during outbreaks. In a bid to increase flu vaccination compliance some health-care institutions have provided HCWs with a voluntary, free immunization program [13, 64]. In these instances, increased rates of HCW vaccination in health care facilities has been associated with a significant reduction in the rate of nosocomial influenza infections amongst patients and staff [13, 56, 64–66].

Limitations of study include an incomplete epidemiological analysis of study patients to account for patient movements within the hospital. The epidemiological observations excluded five of the 14 RXH patients of which two were potential HAIs. Patients 1.1 c and 2.1 b developed infection symptoms within the RXH ICU at nine and 17 days post hospital admission, respectively. Influenza PCR-based testing was unable to determine whether they acquired the infection within this ward. However, access to this information would not have aided our ability to establish virus transmission linkage. Furthermore, the identification of HAI sources may ultimately require exhaustive longitudinal sampling and screening of hospital staff, visitors and patients, including review of patient records and collection of other epidemiological data on questionnaires throughout the duration of a single influenza season. Such a large-scale study would also likely benefit from analyses of larger sequence fragments (possibly even multiple full genome sequences from individual patients at each sampling time-point), which would provide greater analytical power with respect to resolving clusters within phylogenetic trees for the identification and characterization of likely virus transmission chains within the hospital setting.

## Supporting Information

S1 Fig Viral Watch 2011 Western Cape Influenza detection. (PNG)

S1 Table Accession numbers. (PDF)

## Abbreviations

HCW: Health Care Worker
CAI: Community Acquired Infection
HAI: Hospital Acquired Infection
HA: Hemagglutinin
NA: Neuraminidase
BEAST: Bayesian Evolutionary Analysis Sampling Trees
ILI: Influenza-Like Illness
RXH: Red Cross War Memorial Hospital
H1N1: influenza A, Pandemic Subtype H1N1
ML: Maximum Likelihood
MCMC: Markov Chain Monte Carlo
MCC: Maximum Clade Credibility
MRCA: Most recent Common Ancestor
tMRCA: Time to Most Recent Common Ancestor
