# Supplementary figures and images for: Phylogenetic Exploration of Nosocomial Transmission Chains of 2009 Influenza A/H1N1 among Children Admitted at Red Cross War Memorial Children’s Hospital, Cape Town, South Africa in 2011
Source: PLoS One. 2015 Nov 13;10(11):e0141744. doi: 10.1371/journal.pone.0141744 (PMC4643913; doi:10.1371/journal.pone.0141744)

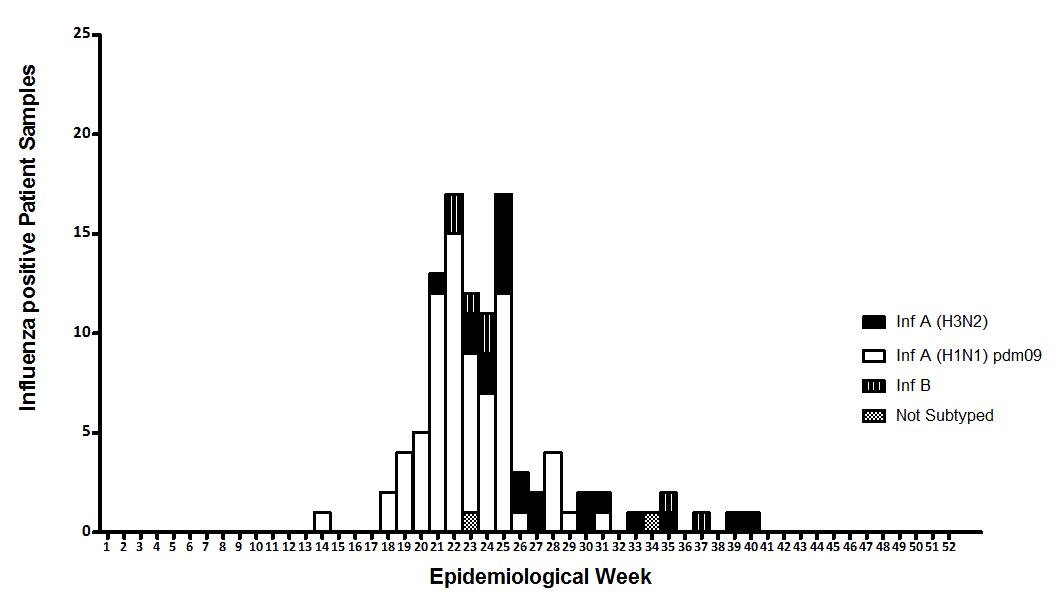

Supplement: S1 Fig — (PNG) [file pone.0141744.s001.png]
