# Supplementary material for: Phylogenetic Exploration of Nosocomial Transmission Chains of 2009 Influenza A/H1N1 among Children Admitted at Red Cross War Memorial Children’s Hospital, Cape Town, South Africa in 2011
Source: PLoS One. 2015 Nov 13;10(11):e0141744. doi: 10.1371/journal.pone.0141744 (PMC4643913; doi:10.1371/journal.pone.0141744)
Supplement: S1 Table — (PDF) [file pone.0141744.s002.pdf]

S1 Table. Accession numbers. Accession numbers and geographical source of study and reference sequences retrieved from NCBI genebank database.

Accession	Location
KC882232	Alabama
KC842172	Alborz
KC881876	Arizona
CY129475	Athens
CY129590	Bonn
CY134465	Boston
CY097972	Budapest
KC020364	Cambodia
JN561789	China
JF327364	Denmark
CY090055	Ecuador
JX413801	Freiburg
JF929764	Guangdong
KC881919	Hawaii
JN790353	Hokkaido
JQ319658	India
CY084210	Korea
CY129990	Kowloon
CY129734	Madrid
JQ914823	Maracay
KC190051	Matara
CY050198	Mexico
CY089387	Mexico
JF801861	Milan
CY099996	Rio Grande
CY099997	Rio Grande
CY099998	Rio Grande
CY091597	Saint-Petersburg
CY129435	Sheffield
JQ065248	Shenzhen
JQ065286	Shenzhen
CY124718	Singapore
JQ693687	Taiwan
